# Different Methods of Physical Training Applied to Women Breast Cancer Survivors: A Systematic Review

**DOI:** 10.3389/fphys.2021.639406

**Published:** 2021-04-14

**Authors:** Silvia Schutz, Felipe J. Aidar, Rafael Luiz Mesquita Souza, Jymmys Lopes dos Santos, Fabrício Azevedo Voltarelli, Roberto Carlos Vieira Junior, Nara Michelle Moura Soares, Anderson Carlos Marçal

**Affiliations:** ^1^Department of Physical Education, Universidade Federal de Sergipe, São Cristóvão, Brazil; ^2^Group of Studies and Research of Performance, Sport, Health, and Paralympic Sports, Universidade Federal de Sergipe, São Cristóvão, Brazil; ^3^Department of Physiology, Universidade Federal de Sergipe, São Cristóvão, Brazil; ^4^Graduate Program of Health Sciences, Faculty of Medicine, Universidade Federal de Mato Grosso, Cuiabá, Brazil; ^5^Coordenação de Educação Física, Universidade Tiradentes, Aracaju, Brazil; ^6^Department of Morphology, Universidade Federal de Sergipe, São Cristóvão, Brazil

**Keywords:** breast tumor, women, physical activity, physical exercise, quality of life

## Abstract

**Objective:** The objective of this systematic review was to identify the effects of different training methods in women who have survived breast cancer (WSBC).

**Data Sources:** Studies were identified by searching SportDiscus, Web of Science, PubMed, Scopus, Scielo, and Bireme.

**Study Selection:** The inclusion criteria were articles that addressed only breast cancer in women, were randomized clinical trials, and interventions involving physical training with Consort ≥80.

**Data Extraction:** The PICO and CONSORT strategies were used for the selection of articles and quality assessment of randomized clinical trials, respectively. Two independent reviewers searched for articles among the databases. Disagreements were discussed, and in the case of an impasse, a third reviewer was consulted.

**Data Synthesis:** Evidence that demonstrated the beneficial effects of physical exercise programs carried out by WSBC. Moderate or high-intensity exercise sessions have been shown to benefit women survivors of breast cancer. Among the modalities, the resistance exercise showed effects from 55% of one-repetition maximum (1 RM), exclusively or associated with other training regimes, such as aerobic (from 48% of heart rate), high-intensity interval training (HIIT), or impact. The main benefits include increased muscle strength, promoted by the practice of resistance exercise in combination with other types of exercises or alone; decreased fatigue; improved quality of life; improved psychosocial effects, and increased leisure time.

**Conclusions:** Physical training performed at a moderate or high intensity (aerobic or anaerobic) can reduce fatigue, improve quality of life, improve sleep quality, and increase bone mineral density in women survivors of breast cancer.

## Introduction

Cancer is one of the main public health problems in different countries. This disease was responsible for the death of ~9.5 million people in 2018 (excluding non-melanoma skin cancer) and is considered the second leading cause of death in the world (Gray et al., 2017; Lewandowska et al., 2019; Wilson et al., 2019). Among the different types of cancer, breast cancer is second most common in the world; it is more common in women and is the type of cancer that causes the most deaths in this population (Harbeck et al., 2019; López-Cortés et al., 2020); moreover, by 2040, there are expected to be ~2,833,941 new cases (Willams et al., 2019; Wilson et al., 2019; Wild et al., 2020).

The incidence of breast cancer is associated with risk factors, such as genetic predisposition, the consumption of alcoholic beverages and tobacco, exposure to estrogen during the use of hormone therapy, and the early use of oral contraceptive methods (Sun et al., 2017). However, other factors, such as older age, benign proliferative breast disease, increased breast density, and radiation exposure, as well as obesity and low levels of physical activity, can also contribute to the development of this pathology (Rojas and Stuckey, 2016; Wild et al., 2020).

Among the different risk factors, it is estimated that sedentary behavior, obesity, and physical inactivity in particular account for 20–40% of all cancer cases. However, some authors suggest that these factors are modifiable since regular physical activity prevents the occurrence of several types of cancer, including bladder, colon, endometrium, esophagus, kidney, stomach, and breast cancer (McTiernan et al., 2019; Patel et al., 2019).

For the treatment of breast cancer, some patients undergo chemotherapy, radiation therapy, or hormone therapy. Despite these treatments being effective, they can cause physiological and psychological impairments that affect the quality of life of patients (Kaltsatou et al., 2010; Schmitz et al., 2019). Of these impairments, the most common are pain, decreased cardiac function, body weight gain, sarcopenia, psychological stress, and cancer-related fatigue (Carayol et al., 2019).

Among the different types of interventions, regular physical exercise performed by cancer survivors can be beneficial for physical function, cancer-related fatigue, pain, and muscle strength (Buffart et al., 2018; Mijwel et al., 2018). Such effects are due, in part, to physical exercise leading to improvements in physical fitness, cardiorespiratory function, muscular endurance, and body composition (Campbell et al., 2019).

According to the American College of Sports Medicine (ACSM) guidelines, aerobic training performed by cancer patients can decrease cancer-related fatigue, increase health-related quality of life and physical function, in addition to reducing anxiety, depression and improve sleep quality. In this population, resistance training proved to be beneficial in decreasing fatigue levels, increasing health-related quality of life and physical function, attenuating lymphedema, and improving aspects related to bone health. The combination of aerobic and resistance exercises decreased fatigue, anxiety, and depression, in addition to increasing health-related quality of life and physical function (Campbell et al., 2019).

Currently, the ACSM recommends that aerobic training is the most effective and safe adjuvant for cancer treatment. The recommendation is that aerobic exercise is performed at moderate intensity for 30 min at least three times a week for a minimum period of 8 to 12 weeks. In comparison with aerobic training, resistance training showed similar effects when it was performed at an intensity of at least 60% of a maximum repetition for a minimum of two sets, including 8–15 repetitions, at least twice per week (Campbell et al., 2019).

However, studies investigating the effects of physical training in women with breast cancer are still scarce. Thus, the present study aimed to gather the scientific evidence that demonstrates the effects of different continuous/regular exercise programs in women who have survived breast cancer (WSBC).

## Materials and Methods

### Literature Research Strategy

The study is a systematic review for which the PICO (**P**atient, **I**ntervention, **C**omparison and **O**utcomes) where: **P** was equivalent to women who survived breast cancer; **I** were interventions based on different training methods; **C** were the comparisons between control and intervention groups and; **O** the outcomes and/or results on the clinical aspects in the health of women survivors of breast cancer. The **PICO** strategy covers a larger number of articles and is recommended when searches are made in a variety of databases (Methley et al., 2014), this strategy was used according to the methodology of Preferred Report Items for Systematic Reviews and Meta-analyzes (PRISMA), considered relevant for the construction of systematic reviews (Moher et al., 2009b).

The SportDiscus, Web of Science, PubMed, Scopus, Scielo, and Bireme databases were searched for relevant articles by two researchers. Only articles published in English, Portuguese or Spanish were included; the keywords used were “aerobic exercise,” “breast cancer,” “breast tumor,” “breast,” “endurance exercise,” “physical exercise,” “females,” “girl,” “interval exercise,” “isometric exercise,” “physical activity,” “resistance exercise,” “strength exercise,” “woman” and “women,” which were crossed with the Boolean operators AND, OR or both operators. Articles published before September 2019 were included.

Studies that included WSBC who performed regular physical exercise and assessed its effects on health were searched, and only randomized clinical trials were selected.

### Inclusion and Exclusion Criteria

The exclusion criteria were as follows: case-control studies, cross-sectional studies, cohort studies, meta-analyses, studies that did not exclusively involve breast cancer, studies that included men in the study population, studies involving specific minorities, studies that did not include physical exercise as an intervention, and studies of experimental models. The inclusion criteria were articles that addressed only breast cancer, included only women, were randomized clinical trials and included interventions involving physical training.

This research assessed the differences between intervention and control groups in terms of bone mineral density, muscle strength, fatigue, role function, quality of life and sleep quality, the maintenance of peak oxygen consumption (peak VO_2_), body composition, and body weight.

### Data Extraction

The Kappa test was used to verify the level of agreement between the reviewers. Two independent reviewers searched for articles among the databases, screened the titles and abstracts, and evaluated the full texts of articles and their eligibility for inclusion in this systematic review. Disagreements were discussed, and in the case of an impasse, a third reviewer was consulted.

Spreadsheets were created according to CONSORT guidelines, and the reviewers used these spreadsheets to extract information about the characteristics, study population, eligibility criteria, intervention methods, and the results reported (Shulz et al., 2010; Falci and Marques, 2015). Each article was reviewed twice by the researchers to guarantee the reliability of the results (Moher et al., 2009a).

### Quality Assessment of Individual Studies

The quality of original works was assessed carefully based on the physical training results in WSBC. Articles with a low risk of bias were used (percentual ≥ 80), as determined by the CONSORT guidelines; methodological quality, as well as the inclusion and exclusion criteria, statistical data, and results, was also verified^1^ (Shulz et al., 2010; Falci and Marques, 2015).

## Results

### Inclusion of Studies

**Figure 1:**
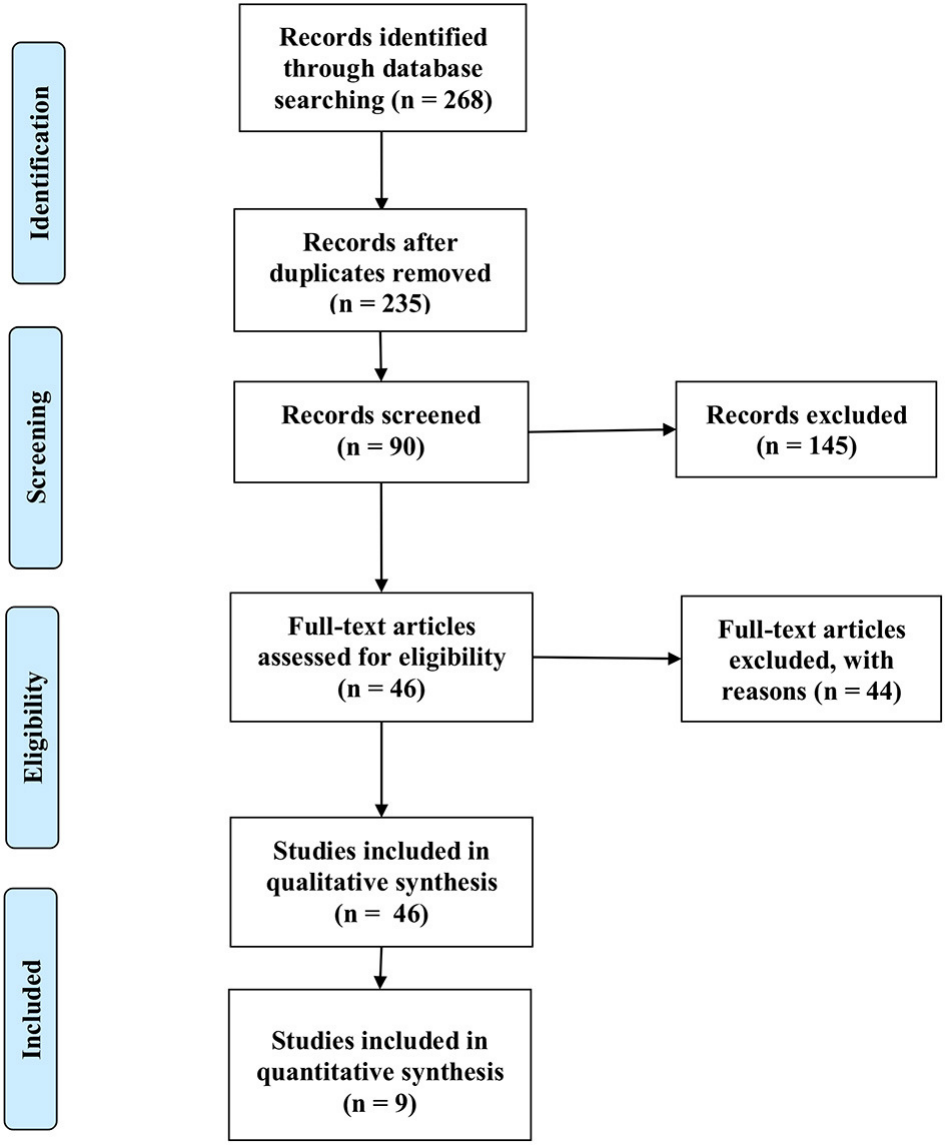
Flow of identification, screening, and eligibility of articles in this systematic review. From Moher et al., 2009a.

After the databases were searched, articles were identified, of which were excluded based on the titles; thus, articles remained eligible for abstract screening. Of these articles, articles were eligible for full-text screening. Finally, nine articles were remaining thatmet the CONSORT inclusion criteria (≥ 80) (Figure 1). The articles were organized in alphabetical order by the surname of the first author (Table 1).

**Table 1:** Main characteristics of the studies included in this review.

**References**	**Participants**	**Intervention**	**Results**	**% Of quality according to consort**
Bloomquist et al. (2019)	153 women after mastectomy, $\bar{\text{x}}$ age 51,7 years old, ongoing chemotherapy, cancer stage: 1-3	**Control group–low** • *Intervention:* walking program, based on a pedometer and an individual consultation. • *Duration:* 12 weeks **Intervention group - high** • *High – until the 6th week*: • *Intervention:* combination of aerobic and resistance exercises • *Intensity:* low and high • *Duration:* 12 weeks *High – until the 12th week*: • *Intervention:* aerobic warm-up, followed by resistance exercises and 15 to 30 min of interval cardiovascular training on stationary bikes • *Intensity:* moderate to high • *Duration:* 12 weeks	↓ Of the BMI ↓ In breast symptoms and in the arms ↑ MS at the extremity of the upper limbs ↓ of pain = Lymphedema = Volume between arms = QV	84
Cormie et al. (2013)	62 women, most underwent mastectomy age 56.1 years, radiation therapy and chemotherapy in ongoing cancer stage	**Control group – UC** • *Intervention:* usual care **Intervention group - High-load** • *Intervention:* resisted exercises • *Intensity:* 75 to 85% of 1 RM using 10 to 6 maximum repetitions • *Frequency:* twice a week (60 min) • *Duration:* 3 months **Intervention group - Low-load** • *Intervention: resisted exercises* • *Intensity:* 55 to 65% of 1 RM using 15 to 20 repetitions • *Frequency:* twice a week (60 min) • *Duration:* 3 months	↑ Physical functioning (QV) = Body pain (QV) = General health (QV) = Vitality (QV) = Social functioning (QV) = Emotional role (QV) = Mental health (QV) = Physical health compound (QV) ↑ Shoulder range of motion = MGF ↑ MS ↑ Muscle endurance = Extent of swelling (lymphedema) = Severity of lymphedema symptoms = Physical function	92
Hagstrom et al. (2015)	39 women after mastectomy $\bar{\text{x}}$ age 51.9 years, chemotherapy, or radiotherapy ongoing cancer stage: 1–3	**Control group:** • Usual medical care. **Intervention group – RT** • *Intervention:* Resistance training • *Intensity:* high • *Frequency*: 3 times a week • *Duration:* 8 weeks	↑ Leisure time ↓ Fatigue ↑ Upper and lower body MS ↑ General QV ↑ Physical well-being (QV) = Functional well-being (QV) = Social well-being (QV) = Emotional well-being (QV)	81
Mijwel et al. (2017)	206 women $\bar{\text{x}}$ age of groups: HIIT = 52.7 years; AT-HIIT = 54.4 years; UC = 52.6 years cancer stage: 1–3, ongoing chemotherapy	**Control group – UC** • *Intervention:* written information about physical activity • *Duration:* 16 weeks **Intervention group - RT-HIIT** • *Intervention:* resistance training and HIIT on a cycle ergometer. • *Intensity:* high • *Duration:* 60 min • *Frequency:* twice a week / 16 weeks **Intervention group - AT-HIIT** • *Intervention:* 20 min of aerobic on a cycle ergometer, elliptical ergometer, or treadmill, followed by HIIT. • *Intensity:* moderate and high • *Duration:* 16 weeks • *Frequency:* twice a week/16 weeks	↑ CRF in the UC group Maintenance of CRF levels in Other groups. ↑ Role function (QV) ↑ Of HRQL ↓ Load of breast cancer symptoms	89
Mijwel et al. (2018)	206 women after mastectomy $\bar{\text{x}}$ age of 52.6 years, cancer stages: 1–3 ongoing chemotherapy	**Control – UC** • *Intervention:* written information about physical activity • *Duration:* 16 weeks **Intervention group - RT-HIIT** • *Intervention:* resistance training directed to the main muscle groups and HIIT in a cycle ergometer. • *Intensity:* high • *Duration:* 60 min • *Frequency:* twice a week/16 weeks **Intervention group - AT-HIIT** • *Intervention:* 20 min aerobic on a cycle ergometer, elliptical ergometer, or treadmill, followed by HIIT. • *Intensity:* moderate and high • *Duration:* 16 weeks • *Frequency:* twice a week/16 weeks	↑ MS ↑ MGF ↓ Threshold PPT Maintaining the CF Maintaining body weight Prevented hyperalgesia ↓ Hemoglobin in all groups Weak inverse R between change in self-reported CRF and change in lower limb strength Inverse R between the change in SRF and the change in PPT in the gluteal muscle‘ No association between change in SRF and change in handgrip strength No association between change in SRF and self-reported change in CF. R between changes in MS of the lower limbs and changes in PPT in the trapezius and glutes, as well as between changes in handgrip and change in PPT in the trapezius. SRF was associated with self-reported pain.	84
Rogers et al. (2015)	42 women after mastectomy $\bar{\text{x}}$ age of 56.2 ongoing chemotherapy or radiation therapy cancer stage: 0–2	**Control group** • Accelerometer monitoring **Intervention group** • *Intervention:* walking and resistance bands • *Intensity:* moderate • *Duration:* 3 months • *Frequency:* twice a week	↓ Daytime sleepiness. ↑Of sleep duration (hours per night) = Sleep quality = Sleep disorder = Accelerometer efficiency and PSQI scale = Accelerometer latency and PSQI scale = Sleeping medications = Global PSQI = Sleep dysfunction = Inflammatory markers (interleukins)	84
Schmidt et al. (2014)	95 women after mastectomy $\bar{\text{x}}$ age of 52.7 chemotherapy in ongoing cancer stages: 1–4	**Control group – RC** • *Intervention*: progressive muscle relaxation • *Duration:* 12 weeks • *Frequency:* twice a week/60 min. **Intervention group – RE** • *Intervention:* Resisted exercises • *Intensity:* 60–80% of 1 RM • *Duration:* 12 weeks • *Frequency:* twice a week/60 min.	↑ Functional function (QV) Improvement of psychosocial effects (QV) ↑ Dry mouth feeling (QV) in the EX group = Physical function (QV) = Cognitive function (QV) = Social function (QV) ↓ Total fatigue in patients without social depression (QV) ↓ Physical fatigue in patients without depression = Affective fatigue = Cognitive fatigue = Physical fatigue = Total fatigue = depression ↑ Cognitive performance on EX only ↑ Total, physical, and affective fatigue in the RC group and maintenance in the EX group (thyroxine users) = Cognitive fatigue (thyroxine users) = Fatigue (not thyroxine users) ↓ Fatigue (smokers)	86
Steindorf et al. (2014)	155 women $\bar{\text{x}}$ age of 55.8 years and cancer stage 0–3 with ongoing radiotherapy after mastectomy.	**Control group: RC** • *Intervention:* progressive muscle relaxation. • *Frequency:* twice a week (60 min/session) • *Duration:* 12 weeks **Intervention group - RE** • *Intervention:* resistance exercise • *Intensity:* 60–80% of 1 RM • *Frequency:* twice a week (60 min/session) • *Duration:* 12 weeks	= Global QV ↑ Functional function (QV) ↓ Pain = Emotional function = Social function = Body image = Depression score = Cognitive performance ↓ Reduce total fatigue ↓ Physical fatigue = Affective fatigue = Cognitive fatigue ↑ MS = Frequency of lymphedema reported	81
Winters-Stone et al. (2011)	106 women $\bar{\text{x}}$ age of 62.3 (POWIR) and 62.2 (FLEX) > 1 year after chemotherapy or radiation therapy. cancer stage: 0–3	**Control group – FLEX** • *Intervention:* stretching and relaxation exercises for the entire body in a sitting or lying position. **Intervention group – POWIR** • *Intervention*: resistance exercises and impact training • *Intensity:* 60–70% of 1 RM • *Frequency:* 2 supervised sessions + 1 home session / week (45–60 min) • *Duration:* 12 months	Favorable changes in bone renewal. Maintenance of BMD in the lumbar spine. = BMD at the hip = body fat and % fat ↑ lean mass in the POWIR group that used AI ↑ Osteocalcin in FLEX and stable in POWIR ↓ Deoxypyridinoline cross-links in POWIR = Effect of using AI or SERM on BMD or fat	84

*↑, increase; ↓, decrease; = no significant difference; $\bar{\text{x}}$, mean; QV, component of the quality of life scale; AI, aromatase inhibitor; BMD, bone mineral density; SERM, selective estrogen receptor modulator; vs, “when compared”; HIIT- High-Intensity Interval Training; UC, Usual Care; RT, Resistance Training; AT, Aerobic Training; RC, Relaxation Control; RE, Resistance Exercise; FLEX, Flexibility Training; POWIR, Prevent Osteoporosis With Impact + Resistance; 1 RM, one-repetition maximum; min, minutes; R, correlation; MS, Muscle strength; CRF, Cancer-Related Fatigue; SRF, Self-Reported Fatigue; Assoc, Association; PPT, Pressure Pain Threshold; CF, Cardiorespiratory Fitness; HRQL, Health-Related Quality of Life; MGF, Manual Grip Force*.

### Project and Study Population

The eligible studies (*n* = 09) reported the experimental design, the randomization of individuals for group allocation, an intervention, and control groups; three studies included two intervention groups and a control group.

The participants' age range was 51 to 62 years. The stage of breast cancer ranged from 0 to 4 as a follow: 0 = Cancer has not grown beyond the point of instigation; 1 = Increased in size and spread to the breast fat tissue; 2 = Affects up to three lymph nodes; 3 = Spread to the chest wall; and 4 = From the breast or the lymph nodes it already reaches other organs or bones (metastatic phase) (Nargis et al., 2019). Not all women had lymphedema, but all women were undergoing cancer treatment (chemotherapy or radiation), except for those included in one study (treatment ended more than a year ago) (Winters-Stone et al., 2011).

### Duration and Types of Training That Have to Affect WSBC

Regular physical activity is related to a lower risk of mortality (Kikuchi et al., 2018) and the development of several chronic diseases, such as cardiovascular diseases, diabetes mellitus, high blood pressure (Hansen et al., 2018), cervix cancer, and breast cancer (Warburton and Bredin, 2017).

However, it is necessary to be aware of the purposes of specific physical exercise prescriptions regarding the modality, frequency, intensity, and duration of training. In addition, an individual's general health condition and disease stage must be observed so that the exercise can be individualized, and the intensity should be gradually adjusted to achieve the desired effect (Luan et al., 2019).

In the present systematic review, we found that the duration of the interventions varied from 8 weeks (Hagstrom et al., 2015), 12 weeks (Schmidt et al., 2014,?; Bloomquist et al., 2019), 16 weeks (Mijwel et al., 2017; Campbell et al., 2019), and 3 months (Cormie et al., 2013; Rogers et al., 2015) to 12 months (Winters-Stone et al., 2011). Among the studies, the physical exercise regimes were classified as aerobic, anaerobic, or a combination of these two types of physical exercise.

Among the different types of training regimes, an article used multimodal sessions for 6 weeks as initial training, consisting of low- and high-intensity exercises. After that period, for the following 6 weeks, six resistance exercises were implemented with a load starting at 70% of one-repetition maximum (1 RM) and gym equipment, targeting the main muscle groups associated with other aerobic activities, which were performed on stationary bikes with peak loads equivalent to 85 to 95% of maximum heart rate (Bloomquist et al., 2019).

Two studies used two combinations of training modalities: a. aerobic exercises of moderate-intensity combined with high-intensity interval training (HIIT); b. resistance training combined with HIIT in WSBC (Mijwel et al., 2017; Campbell et al., 2019). Another study used resistance exercises combined with impact training, which consisted of jumps performed with moderate intensity loads (Winters-Stone et al., 2011).

Moderate to high-intensity resistance exercises were also used as an intervention (Steindorf et al., 2014; Hagstrom et al., 2015; Cešeiko et al., 2019). Additionally, two types of interventions based on resistance exercises were implemented, which differed only in terms of the intensity (low and high); they were compared with each other, and the intensity was inversely proportionally related to the number of repetitions (Cormie et al., 2013).

Another intervention employed was guided aerobic exercise (walking) at moderate intensity combined with strength exercises (resistance bands) and unsupervised walking sessions (Rogers et al., 2015).

Concerning the primary objective, there was little variation in the outcomes, which were as follows: the effects of physical exercise on cancer-related lymphedema (Cormie et al., 2013; Bloomquist et al., 2019); the changes in muscle mass and bone mass after an exercise regime (Winters-Stone et al., 2011); sleep quality after physical training (Rogers et al., 2015); beneficial adjustments in muscle strength, cardiorespiratory fitness, pain, and pressure thresholds and body mass in patients with breast cancer during chemotherapy (Campbell et al., 2019); cancer-related fatigue (Mijwel et al., 2017); and cancer-related fatigue and quality of life (examined by three studies) (Schmidt et al., 2014; Steindorf et al., 2014; Hagstrom et al., 2015).

Among the nine selected articles, six presented the following secondary outcomes: muscle strength (Steindorf et al., 2014; Hagstrom et al., 2015; Bloomquist et al., 2019); quality of life (Cormie et al., 2013; Mijwel et al., 2017); lymphedema symptoms (Cormie et al., 2013; Bloomquist et al., 2019); symptoms related to cancer treatment (Mijwel et al., 2017); leisure time (Hagstrom et al., 2015); psychosocial factors (Rogers et al., 2015); depressive symptoms, cognitive function and cardiorespiratory resistance (VO_2_ peak) (Steindorf et al., 2014); the extent of swelling in the treated arm and physical function (Cormie et al., 2013). It is noteworthy that there were no reports of severe adverse effects after the training regimes in any of the studies analyzed.

### Outcomes and Intervention Measures

Outcome and intervention measures were used to verify whether there were changes in the outcomes after the interventions based on physical exercise were performed; thus, they were categorized for better analysis and understanding.

#### Methods Used to Measure Lymphedema, Bone Mineral Density, and Quality of Sleep

The tools used to assess lymphedema included dual-energy X-ray absorptiometry (DXA) and bioimpedance spectroscopy, and the measurements used included the circumference of body segments and the difference in the volume of the affected and unaffected arms, which reflected the amount of extracellular fluid in the arm (Cormie et al., 2013; Bloomquist et al., 2019). The self-reported symptoms of cancer-related lymphedema were obtained by the Numeric Rating Scale (NRS) (Bloomquist et al., 2019), Disability of the Arm, Shoulder, and Hand (DASH) questionnaire, and Brief Pain Inventory (BPI) questionnaire, in addition to the morbidity subscale of the Functional Assessment of Chronic Illness Therapy (FACT-B +4) questionnaire for breast cancer survivors with lymphedema (Cormie et al., 2013).

Bone mineral density, lean bone mass, and fat mass were assessed by DXA, bone turnover was assessed by serum osteocalcin (ng/mL), and demographic and clinical characteristics were obtained by self-report. For chronic medical conditions, the Charlson Comorbidity Index and the concentration of follicle-stimulating hormones (FSHs) were evaluated to determine whether the patients were undergoing menopause (Winters-Stone et al., 2011).

Sleep quality was assessed using the Pittsburgh Sleep Quality Index (PSQI) and Patient-Reported Outcomes Measurement Information (PROMIS®) scale; the latter scale was also used to assess depression, anxiety, and fatigue. These same authors verified the inflammatory mediators (interleukins (IL): −6, −8, −10 and TNF-alpha) in serum samples from fasting patients; body fat was estimated with bioelectrical impedance; social support and enjoyment for physical activity were assessed by a 5-point Likert scale (Rogers et al., 2015).

#### Methods Used to Measure Fatigue, Quality of Life, Depression, Symptoms Caused by Breast Cancer, and Food Intake

Cancer-related fatigue was assessed using the following questionnaires and scales: Functional Assessment of Cancer Therapy – Fatigue scale (FACIT fatigue) (Hagstrom et al., 2015); Fatigue Assessment Questionnaire (FAQ) (Schmidt et al., 2014; Steindorf et al., 2014); and the Swedish version of the 22-item Piper Fatigue Scale (PFS) (Mijwel et al., 2017; Campbell et al., 2019).

To assess the quality of life, the following questionnaires were used: the European Organization for Research and Treatment of Cancer questionnaire (EORTC-QLQ-C30 version 3.0) (Schmidt et al., 2014; Steindorf et al., 2014; Mijwel et al., 2017; Campbell et al., 2019), the European Organization for Research and Treatment of Cancer questionnaire (EORTC QLQ BR23 version 3.0) (Schmidt et al., 2014; Bloomquist et al., 2019), one of the subscales of these questionnaires (Mijwel et al., 2017), the Functional Assessment of Cancer Therapy – general questionnaire (FACT-G) (Hagstrom et al., 2015) and the Medical Outcomes Study 36-item short-form survey (SF-36) (Cormie et al., 2013).

The symptoms resulting from breast cancer were verified by the Memorial Symptom Assessment Scale (MSAS), which consists of 32 items (Mijwel et al., 2017). Additionally, for the evaluation of depressive symptoms, the 20-item Center for Epidemiologic Studies Depression (CES-D) scale was used.

For cognitive function, the trail-making test was used (Schmidt et al., 2014; Steindorf et al., 2014); leisure time was measured by the Godin leisure-time exercise questionnaire, which categorizes leisure time by three levels of intensity (strenuous, moderate, and light) and evaluates the level of physical activity of the participant over the last 7 days (Hagstrom et al., 2015).

Carbohydrate intake was assessed according to a three-day diet recall (FoodWorks 13) (Rogers et al., 2015); the usual calcium intake (dietary - supplementary) and the total energy intake were assessed using the 2005 Block food frequency questionnaire (Winters-Stone et al., 2011).

#### Methods Used to Measure Patterns of Physical Activity, Muscle Strength, Pain Perception, and Breathing Capacity

An accelerometer was used by the participants to record their physical activity patterns (Rogers et al., 2015; Campbell et al., 2019). The Community Health Activity Model Program for Seniors (CHAMPS) questionnaire for older adults was used (Winters-Stone et al., 2011).

The handgrip strength was verified using a manual hydraulic dynamometer (Cormie et al., 2013; Campbell et al., 2019), muscle strength was measured as the isometric muscle capacity of the thigh and isokinetic strength of representative muscle groups in the upper and lower limbs (Schmidt et al., 2014; Steindorf et al., 2014).

The maximum strength of the upper and lower parts of the body was assessed using 1 RM protocols involving chest presses, seated rows, and leg press exercises (Cormie et al., 2013; Hagstrom et al., 2015; Rogers et al., 2015; Bloomquist et al., 2019). A dynamometer (back and leg dynamometer) was used to evaluate the extensor force of the legs (Rogers et al., 2015).

Hemoglobin and pain were measured, and the latter was assessed bilaterally in the middle trapezius and gluteus muscles with an electronic algometer (Campbell et al., 2019); these same authors assessed cardiorespiratory fitness by the Åstrand-Rhyming submaximal cycle test. However, other authors assessed cardiorespiratory fitness using the submaximal treadmill test (modified Naughton protocol) (Rogers et al., 2015) and resistance performance by the peak VO_2_ and spiroergometric measures (Schmidt et al., 2014) during exercises performed on an exercise bike (Steindorf et al., 2014). The range of motion assessments for the wrist, elbow, and shoulder joints was performed using standard goniometric procedures (Cormie et al., 2013).

### Quality of the Studies

In the evaluation of the individual studies, the agreement between the reviewers was 100% in the analysis of the titles (*k* = 1.00, *p* < 0.001) and 88% in the analysis of abstracts (*K* = 0.88, *p* < 0.001). According to the CONSORT guidelines, out of a total of 46 studies, 13 had scores of <49.9%, 24 studies had scores of 50–79.9%, and nine studies had scores of ≥80%.

Of the 46 articles analyzed, 48% indicated the study model in the title, 35% indicated how the sample size was calculated, 59% described the method used for randomization, 28% reported the adverse effects of the interventions, 35% reported the limitations of the studies, and 47% reported the sources of study funding.

## Discussion

### Overall Outcomes

In this review, evidence that demonstrated the beneficial effects of physical exercise programs carried out by WSBC was gathered from studies considered to be of high quality, so the risk of bias was low (Shulz et al., 2010; Falci and Marques, 2015). Moderate or high-intensity exercise sessions have been shown to benefit WSBC.

The main benefits include increased muscle strength, promoted by the practice of resistance exercise in combination with other types of exercises (Cormie et al., 2013; Hagstrom et al., 2015; Campbell et al., 2019) or alone (Bloomquist et al., 2019); decreased fatigue (Schmidt et al., 2014; Steindorf et al., 2014; Hagstrom et al., 2015); improved quality of life (Steindorf et al., 2014; Mijwel et al., 2017; Bloomquist et al., 2019); improved psychosocial effects (Schmidt et al., 2014) and increased leisure time (Hagstrom et al., 2015).

### Specific Outcomes

#### Changes Promoted by Physical Exercise in Quality of Life, Muscle Strength, and Fatigue in WSBC

Different studies have demonstrated that aerobic exercises combined with resistance exercises at moderate or high intensity are efficient in improving quality of life (Hong et al., 2019), muscle strength (Buffart et al., 2018), and fatigue (Dieli-Conwright et al., 2018).

Muscle function is affected by cancer treatment, in part due to the loss of muscle mass as a consequence of movement limitations and reduced force-generating capacities of muscles (Klassen et al., 2017). However, physical exercises, when practiced during treatment, are effective in maintaining muscle strength (Methley et al., 2014). The exclusive practice of resistance exercise led to significant improvements in muscle strength in the upper body (Cormie et al., 2013) and lower limbs (Hagstrom et al., 2015).

In similar studies, an increase in the muscle strength of WSBC was found after 12 weeks of high-intensity resistance exercise (Cešeiko et al., 2019; Santagnello et al., 2020). Similar results were observed in elderly survivors of BC who performed 16 weeks of resistance exercise at a high intensity (Serra et al., 2018).

When it was performed in combination with other exercises of moderate (walking) and/or high (HIIT) intensities, resistance exercise also improved muscle strength in the upper and lower body (Travier et al., 2015; van Waart et al., 2015).

Cancer-related fatigue is different from that experienced by healthy individuals daily, as it is not relieved with rest nor is it proportional to the level of physical activity performed; thus, it affects patients' quality of life (Berger et al., 2015). In cancer patients, fatigue is a distressing, constant, and subjective symptom of physical, emotional, and/or cognitive tiredness or exhaustion (Bower, 2019). Also, fatigue is one of the symptoms resulting from cancer or its treatment that affects patients with BC and gynecological cancer and is considered one of the main factors for the poor quality of life (Wang and Woodruff, 2015; van Vulpen et al., 2016). Some authors suggested that cancer-related fatigue is due in part to muscular and mitochondrial dysfunction, peripheral immune activation and inflammation dysfunction, as well as central nervous system (CNS) disorder (Yang et al., 2019).

The mechanisms which explain how physical training attenuates cancer-related fatigue are still not entirely clear (Juvet et al., 2017). However, one of the hypotheses suggests that physical training increases functionality, causing a decrease in the physical effort employed and consequently decreasing fatigue (Furmaniak et al., 2016). Another study attests that women breast cancer survivors who participated in a physical training program reported an increased feeling of energy and vigor, which are central aspects of fatigue; thus, physical exercise would be able to decrease the levels of fatigue in this population (Johnsson et al., 2019).

Resistance training, when it is performed in combination with aerobic training, reduced fatigue in women with BC during treatment (van Waart et al., 2015). Similarly, another study (Dieli-Conwright et al., 2018) reported that after 16 weeks of resistance training (intensity 80% of 1 RM for the lower body and 60% for the upper body at 65–80% of the maximum heart rate) reduced the fatigue of WSBC with obesity or overweight who were physically inactive (Dieli-Conwright et al., 2018). In addition, 12 weeks of high-intensity resistance training also decreased fatigue in WSBCs (Santagnello et al., 2020). The same result was detected in elderly survivors of BC when they performed resistance training but at moderate intensity (Serra et al., 2018).

In another study, fatigue levels were higher in sedentary women who were undergoing treatment for BC than in patients who underwent 18 weeks of training involving both resistance and aerobic exercises immediately after treatment for BC (Travier et al., 2015).

Previous meta-analyses, which analyzed physical exercise in different prescription parameters (regardless of whether it was aerobic or anaerobic), demonstrated that training a. reduced fatigue in WSBC (Juvet et al., 2017) who underwent adjuvant therapy (radiation therapy) for the treatment of cancer (Lipsett et al., 2017) and b. improved their quality of life (Meneses-Echávez et al., 2015).

Quality of life can be defined as an individual's well-being, concerning his or her state of mental and physical health, as well as social relationships and economic and environmental factors (Kolotin and Andersen, 2017). In WSBC, this variable can be assessed during the treatment of the disease (Shafaee et al., 2018). Quality of life is strongly affected by the treatment of BC (Chrischilles et al., 2019). However, some authors have suggested that physical exercise promotes significant positive changes in the quality of life and well-being of WSBC (Duncan et al., 2017; Möller et al., 2019).

A resistance training regime of moderate-intensity, not performed in combination with other types of physical exercise, improved the quality of life of elderly survivors of BC (Serra et al., 2018). The combination of both resistance and aerobic training performed at intensities above 60% of the 1 RM and maximum heart rate improved the quality of life of WSBC who were overweight or obese (Dieli-Conwright et al., 2018). In addition, 9 weeks of resistance exercises combined with aerobic exercises positively affected the quality of life and body composition increased the lean mass and decreased the percentage of fat and BMI of WSBC who were being treated with aromatase inhibitors (IAs) (Thomas et al., 2017; Paulo et al., 2018, 2019).

#### Changes in Body Composition and Sleep Quality Promoted by Physical Exercise in WSBC

It is common for individuals affected by BC to become obese after diagnosis, and obesity is associated with worse survival than is the normal weight (Linge et al., 2018; Trestini et al., 2018). However, physical exercise can improve the body composition of cancer survivors (Schwartz et al., 2017).

In one study, sedentary women who underwent BC treatment showed increased body fat and decreased lean mass (Freedman et al., 2004). However, combined training (aerobic exercise and resistance exercise) enhanced lean body mass associate with a reduced percentage of body fat (Thomas et al., 2017). In another study, an increase in muscle strength was found in the extremities of the upper limbs (Bloomquist et al., 2019). Combined training at moderate intensity alleviated symptoms caused by cancer treatment, such as nausea, vomiting, pain, and constipation, in WSBC (van Waart et al., 2015).

The lean mass of WSBC increased after 12 weeks of high-intensity resistance training (Santagnello et al., 2020). After an 11-week intervention involving aerobic and resistance exercises combined with various activities, which involved hypopressive exercises, the BMI of WSBC stabilized, their percentage of body fat decreased (Leclerc et al., 2017).

Lymphedema is the result of the exacerbated retention of lymphatic fluid in the interstitial compartment associated with deficient lymphatic drainage, which can be caused by lymphatic vascular changes, an underlying disease, trauma, or systemic surgery (Grada and Phillips, 2017). Lymphedema impairs the quality of life of WSBC, as it decreases the function of the affected limb (Nelson, 2016; Shah et al., 2016).

Studies have shown that resistance exercise does not increase the extent of swelling and did not worsen symptoms in WSBC with lymphedema (Cormie et al., 2013; Bauman et al., 2018). However, another study also found that resistance exercises both decrease symptoms related to lymphedema and reduces the volume of the WSBC arm (Panchik et al., 2019).

Especially in advanced stages of cancer, patients' sleep and wake cycles are affected (Bernatchez et al., 2017), suggesting that sleep quality may be altered in WSBC. On the other hand, studies have reported that physical exercise can improve sleep quality in WSBC (Matthews et al., 2018; Fang et al., 2019; Kreutz et al., 2019). Accordingly, combined training is known to be more efficient in improving the quality of sleep and the number of hours of sleep per night, as combined training helps reduces the severity of sleep disorders and daytime sleepiness (Rogers et al., 2015). Also, resistance training for 12 weeks, at an intensity of 60–80% of 1 RM, decreased sleep disorders in WSBC (Steindorf et al., 2017).

#### Changes in Cardiorespiratory Fitness Promoted by Physical Exercise in WSBC

Cardiorespiratory fitness is considered both an indicator of an individual's health and the prognoses of diseases (Ozemec et al., 2018). Some authors have suggested that physical exercise is an effective means of improving cardiorespiratory fitness and decreasing cardiometabolic risk in individuals with pathological conditions (Ruegsegger and Booth, 2018).

In WSBC, physical exercise can improve cardiorespiratory responses and reduce fatigue (Ruegsegger and Booth, 2018). In a previous study (Campbell et al., 2019), WSBC showed an increase in muscle strength and handgrip strength and maintained the same level of cardiorespiratory fitness and body weight after resistance and combined training, both of which include components of HIIT. On the other hand, in the same study (Campbell et al., 2019), in the control group, such responses were not observed. In another study (Mijwel et al., 2017), WSBC maintained similar fatigue scores and showed improved role function scores on the quality of life scale (EORTC-QLQC30) after combined training; such positive responses were not observed in the control group (Mijwel et al., 2017). Nevertheless, combined training maintained the cardiorespiratory fitness of WSBC; however, the sedentary group showed worse cardiorespiratory fitness (van Waart et al., 2015).

Eighteen weeks of combined training, including HIIT components, improved the submaximal cardiorespiratory fitness of WSBC (Travier et al., 2015). Another work (Dieli-Conwright et al., 2018) showed that overweight/obese WSBC who underwent 16 weeks of combined training (60% of the 1 RM for the upper body and 80% for the lower body in resistance exercises and 65–80% of the maximum heart rate for aerobic exercises) showed improvement in cardiorespiratory fitness.

In addition, the combination of combined training and flexibility exercises for 12 weeks improved pain perception, maximum VO_2_, flexibility, and muscle strength in WSBC (Reis et al., 2018); similar results, as well as improvements in shoulder pain, were observed in another study after combined training (Möller et al., 2019).

#### Changes in Bone Mineral Density Promoted by Physical Exercise in WSBC

In women diagnosed with BC, treatment with AIs is considered the standard pharmacological method to increase patient survival (Geisler, 2011). However, these drugs have the side effects of increased bone resorption (Baker et al., 2018), accelerated bone loss, and, consequently, an increased risk of fractures (Ramchand et al., 2019).

One method used to stimulate bone remodeling and increase bone mineral density is the practice of impact physical training, which must be performed with loads greater than that experienced in day-to-day life (Kirkham et al., 2016). In addition to the appropriate intensity, adherence to the physical exercise program is essential to improve bone mineral density in WSBC (Kemmler et al., 2016).

In the other study, women with BC being treated with IAs showed favorable changes in bone renewal, the maintenance of bone mineral density in the spine and lumbar spine as well as an increase in lean mass after 12 months of impact training combined with resistance exercises (Winters-Stone et al., 2011). In another study (Zaidi et al., 2018), women with BC using IAs were able to maintain their total bone mineral density after performing aerobic training; in the group that performed impact training combined with resistance exercise, the bone mineral density in the patients' spine was preserved (Zaidi et al., 2018).

#### Weaknesses Detected During the Analysis of the Articles and Suggestions for Future Studies

Before listing the gaps found in some studies, we must first emphasize that each selected work has important strengths that contributed to improving the understanding of the effects of physical training in women with BC for the scientific and academic community. Thus, at no time did we aim to undermine the efforts of the authors of the scientific articles referenced in this systematic review.

The most common problems were related to the data collection of several parameters inherent for the control groups in the studies. Among the articles selected for this systematic review, we detected the absence of a sedentary group, as the control groups did not undergo any interventions and/or performed stretching exercises (Winters-Stone et al., 2011). Also, another important point was that aerobically active women were not excluded, 62% of whom adhered to the interventions. In this same study, the training program lasted 12 months, with the ideal period being longer for the completion of bone remodeling cycles.

The daily activities of the participants who did not undergo a physical exercise intervention also involved an individualized walking program (monitoring by a pedometer) and were encouraged to progressively increase the number of steps they took each day until they reached 10,000 steps per day (Bloomquist et al., 2019). Furthermore, in the same study (Bloomquist et al., 2019), lymphedema of the upper limbs after resistance exercise was not assessed, and the women who did not undergo training had more baseline-related lymphedema than did those who underwent physical training; nevertheless, we noticed the absence of data on the volume between the arms and the measurements of extracellular volume in all patients (Bloomquist et al., 2019).

In another study, the participants were not evenly distributed among groups during the training period, and we also detected (a) the use of broad recruitment criteria; (b) a lack of limits to restrict the variation in the time after the treatment of BC; (c) the failure to perform power calculations for secondary purposes and assess the effects on fatigue and muscle strength in the treated limb; (d) insufficient data from the intervention group to allow an analysis of how social interactions improved due to physical exercise (Hagstrom et al., 2015). We emphasize that the motivational aspect of patients participating in the study can be a limiting agent of the research since it does not represent the behavior of all women with lymphedema related to BC or even the use of compression clothes.

In other studies, the sample sizes were relatively small for subgroup analyzes (Cormie et al., 2013), and a large number of patients randomized to the control group did not participate in the research (Campbell et al., 2019) and had a high rate of abandonment of physical training (Mijwel et al., 2017).

In another study, limitations such as a small sample size, short exercise sessions, and a lack of assessments for more detailed aspects of sleep in patients with BC were detected; moreover, in that same study, it was not clear whether the results can be generalized to other types of cancer, and the effects of exercise on psychosocial factors were not distinguished (Rogers et al., 2015).

Concerning the research participants, not all studies verified their daily physical activity routines (Cormie et al., 2013; Steindorf et al., 2014; Hagstrom et al., 2015; Rogers et al., 2015; Bloomquist et al., 2019), and information on food, alcohol intake, and/or tobacco use was not evaluated (Winters-Stone et al., 2011; Cormie et al., 2013; Schmidt et al., 2014; Hagstrom et al., 2015; Mijwel et al., 2017; Bloomquist et al., 2019; Campbell et al., 2019).

### Study Limitations

This systematic review has important strengths, such as a low risk of bias. However, some limitations must be mentioned, such as a small number of articles and strict criteria for the analysis of specific aspects of each type of physical training (load, intensity, and volume). It was found that there is strong evidence showing that regular physical exercise performed at a moderate or high intensity, regardless of whether it is aerobic or anaerobic, alone or in combination with other exercise modalities, can benefit WSBC. Among these benefits, we highlight increased muscle strength, improved quality of life, and decreased fatigue. In addition, positive effects on sleep quality as well as the maintenance of bone density and the bone turnover rate were observed. However, in future studies, it is necessary to individualize the WSBC regarding the type of treatment, stages of the disease and time after the diagnosis of breast cancer, and what is the best type of training that they should carry out to improve health and quality of life.

## Conclusions

We recommend that the regular practice of physical exercise, supervised and prescribed by the doctor of the patient, is important for the maintenance and/or recovery of the health and quality of life of WSBC.

Physical training programs that include resistance exercise exclusively at an intensity of 55–80% performed at least twice a week and targeted at the main muscle groups have proven to be efficient in improving parameters related to the quality of life, muscle strength, endurance, physical function, cognitive performance, and leisure time; also, decreased levels of fatigue and maintenance of lymphedema status can be verified.

The combination of physical exercises, such as aerobic (85–95% of maximum heart rate) associated with resistance (70–90% of 1 RM) decreased BMI, breast and arm symptoms, and pain, in addition to increasing muscle strength. HIIT associated with resistance training, with an intensity of 70–80% of 1 RM; or HIIT plus aerobic exercise (with a score of 13–15 on Borg's perceived exertion scale) provided positive effects on quality of life, muscle strength, and handgrip, body weight stabilization and fatigue in addition to reducing pain and symptoms related to breast cancer.

A physical exercise program that made use of resistance bands associated with walking (48–52% of heart rate) for 3 months brought benefits on the quality of sleep of breast cancer survivors, such as decreased daytime sleepiness and increased number of hours of sleep per night. Impact exercises (jumping with heavy vests) combined with resistance exercises, with an intensity of 60–70% of 1 RM, promoted favorable bone changes as well as an increase in lean mass in women survivors of breast cancer who used IAS.

## Data Availability Statement

The original contributions generated for the study are included in the article/Supplementary Material, further inquiries can be directed to the corresponding author/s.

## Author Contributions

SS and RS: conceptualization, data treatment, and wrote – original draft. SS, NS, and AM: formal analysis. SS, RS, and FV: investigation. NS: methodology. NS and AM: project administration. FA, JdS, FV, RV, NS, and AM: wrote – review and editing. All authors contributed to the article and approved the submitted version.

## Conflict of Interest

The authors declare that the research was conducted in the absence of any commercial or financial relationships that could be construed as a potential conflict of interest.

## Footnotes

^1^http://www.consort-statement.org.
